# Generalized $(p,q)$-Bleimann-Butzer-Hahn operators and some approximation results

**DOI:** 10.1186/s13660-017-1582-x

**Published:** 2017-12-19

**Authors:** M Mursaleen, Md Nasiruzzaman, Khursheed J Ansari, Abdullah Alotaibi

**Affiliations:** 1 Department of Mathematics, Aligarh Muslim University, Aligarh, 202002 India; 2 Operator Theory and Applications Research Group, Department of Mathematics, Faculty of Science, King Abdulaziz University, P.O. Box 80203, Jeddah, 21589 Saudi Arabia; 3 Department of Mathematics, Jamia Millia Islamia University, New Delhi, 110005 India; 4 Department of Mathematics, College of Science, King Khalid University, Abha, 61413 Saudi Arabia

**Keywords:** 41A10, 41A25, 41A36, $(p,q)$-integers, $(p,q)$-Bernstein operators, *q*-Bleimann-Butzer-Hahn operators, $(p,q)$-Bleimann-Butzer-Hahn operators, modulus of continuity, rate of approximation, Lipschitz type maximal function space

## Abstract

The aim of this paper is to introduce a new generalization of Bleimann-Butzer-Hahn operators by using $(p,q)$-integers which is based on a continuously differentiable function *μ* on $[0,\infty)=\mathbb{R}_{+}$. We establish the Korovkin type approximation results and compute the degree of approximation by using the modulus of continuity. Moreover, we investigate the shape preserving properties of these operators.

## Introduction and preliminaries

The *q*-generalization of Bernstein polynomials [1] was introduced by Lupaş [2] as follows:

$$ L_{n,q}(f;x)=\frac{1}{\prod_{j=1}^{n}\{(1-x)+q^{j-1}x\}}\sum_{i=0}^{n}f \biggl( \frac{[i]_{q}}{[n]_{q}} \biggr) \left [ \textstyle\begin{array}{@{}c@{}} n \\ i \end{array}\displaystyle \right ] _{q}q^{\frac{i(i-1)}{2}}x^{i}(1-x)^{n-i}. $$

In 1997, Phillips [3] introduced another modification of Bernstein polynomials, obtained the rate of convergence and the Voronovskaja type asymptotic expansion for these polynomials.

The $(p, q)$-integer was introduced in order to generalize or unify several forms of *q*-oscillator algebras well known in the early physics literature related to the representation theory of single parameter quantum algebras [4].

In the recent years, the first $(p,q)$-analogue of Bernstein operators was introduced by Mursaleen et al. (see [5]), and some approximation properties were studied (see [6–9]). Moreover, the $(p,q)$-calculus in computer-aided geometric design (CAGD) given by Khalid et al. (see [10]) will help readers to understand the applications. Besides this, we also refer the reader to some recent papers on $(p,q)$-calculus in approximation theory [11–20] and [21].

We recall some definitions and notations of $(p,q)$-calculus.

The $(p,q)$ integers $[n]_{p,q}$ are defined by

1.1 $$ \begin{aligned} & [n ]_{p,q}=p^{n-1}+qp^{n-2}+ \cdots+q^{n-1}= \textstyle\begin{cases} \frac{p^{n}-q^{n}}{p-q} & (p \neq q \neq1), \\ \frac{1-q^{n}}{1-q} & (p = 1), \\ n & (p = q=1), \end{cases}\displaystyle \\ &(au+bv)_{p,q}^{n}:=\sum_{i=0}^{n}p^{\frac{(n-i)(n-i-1)}{2}}q^{\frac{i(i-1)}{2}} \left [ \textstyle\begin{array}{@{}c@{}} n \\ i \end{array}\displaystyle \right ] _{p,q}a^{n-i}b^{i}u^{n-i}v^{i}, \\ &(u+v)_{p,q}^{n}=(u+v) (pu+qv) \bigl(p^{2}u+q^{2}v \bigr)\cdots\bigl(p^{n-1}u+q^{n-1}v\bigr), \\ &(1-u)_{p,q}^{n}=(1-u) (p-qu) \bigl(p^{2}-q^{2}u \bigr)\cdots\bigl(p^{n-1}-q^{n-1}u\bigr) \end{aligned} $$

and the $(p,q)$-binomial coefficients are defined by

$$ \left [ \textstyle\begin{array}{@{}c@{}} n \\ i\end{array}\displaystyle \right ] _{p,q}= \frac{[n]_{p,q}!}{[i]_{p,q}![n-i]_{p,q}!}. $$

By a simple calculation [5], we have the following relation:

$$ q^{i}[n-i+1]_{p,q}=[n+1]_{p,q}-p^{n-i+1}[i]_{p,q}. $$

For details on *q*-calculus and $(p,q)$-calculus, one can refer to [22–25].

Totik [26] studied the uniform approximation properties of Bleimann-Butzer-Hahn operators [27] when *f* belongs to the class $C(\mathbb{R}_{+})$ of continuous functions on $\mathbb{R}_{+}$ that have finite limits at infinity.

The Bleimann-Butzer-Hahn operators (BBH) based on *q*-integers are defined as follows:

$$ L_{n}^{q}(f;x)=\frac{1}{\ell_{n,q}(x)}\sum _{i=0}^{n}f \biggl( \frac {[i]_{q}}{[n-i+1]_{q}q^{i}} \biggr) q^{\frac{i(i-1)}{2}}\left [ \textstyle\begin{array}{@{}c@{}} n \\ i\end{array}\displaystyle \right ] _{q}x^{i}, $$

where $\ell_{n,q}(x)=\prod_{i=0}^{n-1}(1+q^{s}x)$. For $q=1$, these operators reduce to the classical BBH operators [27].

For $f\in C[0,1]$, $x\in{}[0,1]$, Morales et al. [28] introduced a new generalization of Bernstein polynomials denoted by $B_{n}^{\mu}$

$$ B_{n}^{\mu}(f;x)=B_{n}\bigl(f\circ \mu^{-1};\mu(x)\bigr)=\sum_{i=0}^{n} \left [ \textstyle\begin{array}{@{}c@{}} n \\ i\end{array}\displaystyle \right ] \mu(x)^{i}\bigl(1- \mu(x)\bigr)^{n-i}\bigl(f\circ\mu^{-1}\bigr) \biggl( \frac {i}{n} \biggr) , $$

where *μ* is a continuously differentiable function of infinite order on $[0,1]$ such that $\mu(0)=0$, $\mu(1)=1$, and $\mu^{\prime}(x)>0$ for $x\in{}[0,1]$. They have also studied some shape preserving and convergence properties on approximation concerning the generalized Bernstein operators $B_{n}^{\mu}(f;x)$.

For $0< q< p\leq1$ and *f* defined on semiaxis $\mathbb{R}_{+}$, we give a generalization of $(p,q)$-Bleimann-Butzer-Hahn type operators (see [21]) as follows:

1.2 $$ L_{n,\mu}^{p,q}(f;x)=\frac{1}{\ell_{n,\mu}^{p,q}(x)}\sum _{i=0}^{n}\bigl(f\circ\mu ^{-1} \bigr) \biggl( \frac{p^{n-i+1}[i]_{p,q}}{[n-i+1]_{p,q}q^{i}} \biggr) p^{\frac{(n-i)(n-i-1)}{2}}q^{\frac{i(i-1)}{2}}\left [ \textstyle\begin{array}{@{}c@{}} n \\ i\end{array}\displaystyle \right ] _{p,q}\mu(x)^{i}, $$

where

$$ \ell_{n,\mu}^{p,q}(x)=\prod_{s=0}^{n-1} \bigl(p^{s}+q^{s}\mu(x)\bigr), $$

and *μ* is a continuously differentiable function defined on $\mathbb {R}_{+}$ having the property

1.3 $$ \mu(0)=0\quad \mbox{and}\quad \inf_{x\in[0,\infty)}\mu^{\prime }(x) \geq1. $$

We can easily see that

$$ L_{n,\mu}^{p,q}f=L_{n,p,q}\bigl(f\circ\mu^{-1} \bigr)\mu, $$

where $L_{n,p,q}$ is defined in [21] as

$$ L_{n,p,q}(f;x)=\frac{1}{\ell_{n,p,q}(x)}\sum_{i=0}^{n}f \biggl( \frac{p^{n-i+1}[i]_{p,q}}{[n-i+1]_{p,q}q^{i}} \biggr) p^{\frac {(n-i)(n-i-1)}{2}}q^{\frac{i(i-1)}{2}}\left [ \textstyle\begin{array}{@{}c@{}} n \\ i\end{array}\displaystyle \right ] _{p,q}x^{i}. $$

The operators defined by (1.2) are more flexible and sensitive to the rate of convergence than the $(p,q)$-BBH operators. Our results show that the new operators are sensitive to the rate of convergence to *f*, depending on the selection of *μ*. For the particular case $\mu(x)=x$, the previous results for $(p,q)$-Bleimann-Butzer-Hahn operators are obtained (see [21]).

### Lemma 1.1

*Let* $L_{n,\mu}^{p,q}$ *be operators defined by* (1.2). *Then*, *for a continuously differentiable function* $\mu(x)$ *on* $\mathbb{R}_{+}$ *defined by* (1.3), *we have*

1.4 $$ L_{n,\mu}^{p,q}(f;x)=\textstyle\begin{cases} 1,& \textit{for } f(t)=1, \\ \frac{p[n]_{p,q}}{[n+1]_{p,q}} ( \frac{\mu(x)}{1+\mu(x)} ) ,& \textit{for } f(t)=\frac{\mu(t)}{1+\mu(t)}, \\ \frac{pq^{2}[n]_{p,q}[n-1]_{p,q}}{[n+1]_{p,q}^{2}}\frac{\mu (x)^{2}}{(1+\mu(x))(p+q\mu(x))}+\frac {p^{n+1}[n]_{p,q}}{[n+1]_{p,q}^{2}} ( \frac{\mu(x)}{1+\mu(x)} ) ,& \textit{for } f(t)= ( \frac{\mu(t)}{1+\mu(t)} ) ^{2}. \end{cases} $$

### Proof

For the proof of this lemma, we refer to [21]. □

## Korovkin type approximation result

Here we propose to obtain a Korovkin type approximation theorem for operators $L_{n,\mu}^{p,q}$.

Let $C_{B}(\mathbb{R}_{+})$ denote the set of all bounded and continuous functions defined on $\mathbb{R}_{+}$. $C_{B}(\mathbb{R}_{+})$ is a normed linear space with

$$ \| f\|_{C_{B}}=\sup_{u\geq0} \bigl\vert f(u) \bigr\vert . $$

The modulus of continuity *ω* is a non-negative and non-decreasing function defined on $\mathbb{R}_{+}$ such that it is sub-additive and $\lim_{\delta\rightarrow0}\omega(\delta)=0$.

One can easily see that

2.1 $$\begin{aligned}& \omega(n\delta)\leq n \omega(\delta), \quad n \in\mathbb{N}, \end{aligned}$$

2.2 $$\begin{aligned}& \omega(\lambda\delta)\leq\omega\bigl(1+\bigl[ \vert \lambda \vert \bigr] \bigr)\leq1+\lambda \omega(\delta), \quad \lambda>0, \end{aligned}$$

where $[|\lambda|]$ denotes the greatest integer which is not greater than *λ*.

Let ${H}_{\omega}$ denote the space of all real-valued functions *f* defined on $\mathbb{R}_{+}$ satisfying

2.3 $$ \bigl\vert f(u) - f(v) \bigr\vert \leq\omega \biggl( \biggl\vert \frac{\mu(u)}{1+\mu(u)}- \frac{\mu(v)}{1+\mu(v)} \biggr\vert \biggr) $$

for any $u,v\in\mathbb{R}_{+}$.

### Theorem 2.1

([29])

*Let* $P_{n}:H_{\omega}\rightarrow C_{B}(\mathbb {R}_{+})$ *be a sequence of positive linear operators such that*

$$ \lim_{n\rightarrow\infty} \biggl\Vert P_{n} \biggl( \biggl( \frac{\mu (t)}{1+\mu (t)} \biggr) ^{\nu};x \biggr) - \biggl( \frac{\mu(x)}{1+\mu(x)} \biggr) ^{\nu } \biggr\Vert _{C_{B}}=0 $$

*for* $\nu=0,1,2$. *Then*, *for any function* $f\in H_{\omega}$,

$$ \lim_{n\rightarrow\infty} \bigl\Vert P_{n}(f)-f \bigr\Vert _{C_{B}}=0. $$

To compute the convergence results for the operators $L_{n,\mu}^{p,q}$ defined by (1.2), we take $q=q_{n}$, $p=p_{n}$, where $0< q_{n}< p_{n}\leq1$ satisfying

2.4 $$\begin{aligned}& \lim_{n}p_{n}=1,\qquad \lim _{n}q_{n}=1, \end{aligned}$$

2.5 $$\begin{aligned}& \lim_{n}p_{n}^{n}=a,\qquad \lim_{n}q_{n}^{n}=b \quad (0< a,b \leq1). \end{aligned}$$

### Theorem 2.2

*Let* $L_{n,\mu}^{p,q}$ *be operators defined by* (1.2) *and take* $p=p_{n}$, $q=q_{n}$ *satisfying* (2.5). *Then*, *for* $0< q_{n}< p_{n}\leq1$ *and any function* $f\in H_{\omega}$, *we have*

2.6 $$ \lim_{n} \bigl\Vert L_{n,\mu}^{p_{n},q_{n}}(f)-f \bigr\Vert _{C_{B}}=0. $$

### Proof

Here we use Theorem 2.1. For $\nu=0,1,2$, it is sufficient to verify the following three conditions:

$$ \lim_{n\rightarrow\infty} \biggl\Vert L_{n,\mu}^{p_{n},q_{n}} \biggl( \biggl( \frac{\mu(t)}{1+\mu(t)} \biggr) ^{\nu};x \biggr) - \biggl( \frac{\mu (x)}{1+\mu(x)} \biggr) ^{\nu} \biggr\Vert _{C_{B}}=0. $$

For $\nu=0$, applying Lemma 1.1, (2.6) is fulfilled. Now, we observe that

$$\begin{aligned}& \biggl\Vert L_{n,\mu}^{p_{n},q_{n}} \biggl( \biggl( \frac{\mu(t)}{1+\mu (t)} \biggr) ;x \biggr) - \biggl( \frac{\mu(x)}{1+\mu(x)} \biggr) \biggr\Vert _{C_{B}} \\& \quad \leq \biggl\vert \frac{p_{n}[n]_{p_{n},q_{n}}}{[n+1]_{p_{n},q_{n}}}-1 \biggr\vert \\& \quad \leq \biggl\vert \biggl( \frac{p_{n}}{q_{n}} \biggr) \biggl( 1-p_{n}^{n}\frac {1}{[n+1]_{p_{n},q_{n}}} \biggr) -1 \biggr\vert . \end{aligned}$$

Here we have used $q_{n}[n]_{p_{n},q_{n}}=[n+1]_{p_{n},q_{n}}-p_{n}^{n}$, $[n+1]_{p_{n},q_{n}} \rightarrow\infty$ as $n\rightarrow\infty$, equation (2.6) holds for $\nu=1$. Now, to verify for $\nu=2$, we see that

$$\begin{aligned} \begin{aligned} &\biggl\Vert L_{n,\mu}^{p_{n},q_{n}} \biggl( \biggl( \frac{\mu(t)}{1+\mu (t)} \biggr) ^{2};x \biggr) - \biggl( \frac{\mu(x)}{1+\mu(x)} \biggr) ^{2} \biggr\Vert _{C_{B}} \\ &\quad =\sup_{x\geq0} \biggl\{ \frac{\mu(x)^{2}}{(1+\mu(x))^{2}} \biggl( \frac{ p_{n}q_{n}^{2}[n]_{p_{n},q_{n}}[n-1]_{p_{n},q_{n}}}{[n+1]_{p_{n},q_{n}}^{2}}\cdot \frac{1+\mu(x)}{p_{n}+q_{n}\mu(x)}-1 \biggr) \\ &\qquad {}+\frac{p_{n}^{n+1}[n]_{p_{n},q_{n}}}{[n+1]_{p_{n},q_{n}}^{2}}\cdot \frac{\mu (x)}{1+\mu(x)} \biggr\} . \end{aligned} \end{aligned}$$

By a simple calculation, we have

$$ \frac{[n]_{p_{n},q_{n}}[n-1]_{p_{n},q_{n}}}{[n+1]_{p_{n},q_{n}}^{2}}= \frac{1}{q_{n}^{3}} \biggl\{ 1-p_{n}^{n} \biggl( 2+\frac{q_{n}}{p_{n}} \biggr) \frac{1}{[n+1]_{p_{n},q_{n}}}+\bigl(p_{n}^{n} \bigr)^{2} \biggl( 1+\frac {q_{n}}{p_{n}} \biggr) \frac{1}{[n+1]_{p_{n},q_{n}}^{2}} \biggr\} , $$

and

$$ \frac{[ n]_{p_{n},q_{n}}}{[n+1]_{p_{n},q_{n}}^{2}}=\frac{1}{q_{n}} \biggl( \frac{1}{[n+1]_{p_{n},q_{n}}}-p_{n}^{n} \frac{1}{[n+1]_{p_{n},q_{n}}^{2}} \biggr) . $$

Thus, we have

$$\begin{aligned}& \biggl\Vert L_{n,\mu}^{p_{n},q_{n}} \biggl( \biggl( \frac{\mu(t)}{1+\mu (t)} \biggr) ^{2};x \biggr) - \biggl( \frac{\mu(x)}{1+\mu(x)} \biggr) ^{2} \biggr\Vert _{C_{B}} \\& \quad \leq\frac{p_{n}}{q_{n}} \biggl\{ 1-p_{n}^{n} \biggl( 2+ \frac{q_{n}}{p_{n}} \biggr) \frac{1}{[n+1]_{p_{n},q_{n}}}+\bigl(p_{n}^{n} \bigr)^{2} \biggl( 1+\frac {q_{n}}{p_{n}} \biggr) \frac{1}{[n+1]_{p_{n},q_{n}}^{2}}-1 \biggr\} \\& \qquad {} +p_{n}^{n}\frac{p_{n}}{q_{n}} \biggl( \frac {1}{[n+1]_{p_{n},q_{n}}}-p_{n}^{n}\frac{1}{[n+1]_{p_{n},q_{n}}^{2}} \biggr) . \end{aligned}$$

Hence (2.6) holds for $\nu=2$, and the proof is completed by Theorem 2.1. □

## Rate of convergence

In this section, we determine the rate of convergence of operators $L_{n,\mu}^{p,q}$.

For $f\in H_{\omega}$, the modulus of continuity is defined by

$$ \widetilde{\omega}(f;\delta)=\sum_{\substack{ |\frac{\mu (u)}{1+\mu(u)}-\frac{\mu(v)}{1+\mu(v)}|\leq\delta, \\ u,v\geq0}} \bigl\vert f(u)-f(v) \bigr\vert $$

which satisfies the following conditions:

1. $\widetilde{\omega}(f;\delta)\rightarrow0$ ($\delta \rightarrow0$);
2. $| f(u)-f(v)|\leq\widetilde{\omega}(f;\delta) ( \frac{|\frac{\mu(u)}{1+\mu(u)}-\frac{\mu(v)}{1+\mu(v)}|}{\delta}+1 )$.

### Theorem 3.1

*Let* $p=p_{n}$, $q=q_{n}$, $0< q_{n}< p_{n}\leq1$ *satisfying* (2.5). *Then*, *for each* *μ* *defined by* (1.3) *on* $\mathbb{R}_{+}$ *and for any function* $f\in H_{\omega}$, *we have*

$$ \bigl\vert L_{n,\mu}^{p_{n},q_{n}}(f;x)-f(x) \bigr\vert \leq2 \widetilde{\omega}\Bigl(f;\sqrt {\delta_{n}^{\mu}(x)} \Bigr), $$

*where*

$$\begin{aligned} \delta_{n}^{\mu}(x) =&\frac{\mu(x)^{2}}{(1+\mu(x))^{2}} \biggl( \frac{p_{n}q_{n}^{2}[n]_{p_{n},q_{n}}[n-1]_{p_{n},q_{n}}}{[n+1]_{p_{n},q_{n}}^{2}} \frac{1+\mu(x)}{p_{n}+q_{n}\mu(x)}-2\frac{p_{n}[n]_{p_{n},q_{n}}}{[n+1]_{p_{n},q_{n}}}+1 \biggr) \\ &{}+ \frac{p_{n}^{n+1}[n]_{p_{n},q_{n}}}{[n+1]_{p_{n},q_{n}}^{2}}\frac{\mu(x)}{1+\mu(x)}. \end{aligned}$$

### Proof

For $L_{n,\mu}^{p_{n},q_{n}}$, we have

$$\begin{aligned} \bigl\vert L_{n,\mu}^{p_{n},q_{n}}(f;x)-f(x)\bigr\vert \leq& L_{n,\mu}^{p_{n},q_{n}} \bigl( \bigl\vert f(t)-f(x) \bigr\vert ;x \bigr) \\ \leq& \widetilde{\omega}(f; \delta) \biggl\{ 1+\frac{1}{\delta} L_{n,\mu}^{p_{n},q_{n}} \biggl( \biggl\vert \frac{\mu(t)}{1+\mu(t)}- \frac{\mu (x)}{1+\mu(x)} \biggr\vert ;x \biggr) \biggr\} . \end{aligned}$$

Applying the Cauchy-Schwarz inequality, we get

$$\begin{aligned}& \bigl\vert L_{n,\mu}^{p_{n},q_{n}}(f;x)-f(x) \bigr\vert \\& \quad \leq \widetilde{\omega}(f; \delta_{n}) \biggl\{ 1+ \frac{1}{\delta_{n}} \biggl[ \biggl( L_{n,\mu}^{p_{n},q_{n}} \biggl( \frac{\mu(t)}{1+\mu(t)}-\frac{\mu(x)}{1+\mu(x)} \biggr)^{2};x \biggr) \biggr]^{\frac{1}{2}} \bigl( L_{n,\mu}^{p_{n},q_{n}}(1;x) \bigr)^{\frac{1}{2}} \biggr\} \\& \quad \leq\widetilde{\omega}(f; \delta_{n}) \biggl\{ 1+ \frac{1}{\delta_{n}} \biggl[ \frac{\mu(x)^{2}}{(1+\mu(x))^{2}} \biggl( \frac{p_{n}q_{n}^{2}[n]_{p_{n},q_{n}}[n-1]_{p_{n},q_{n}}}{[n+1]_{p_{n},q_{n}}^{2}} \frac{1+\mu (x)}{p_{n}+q_{n}\mu(x)} \\& \qquad {}-2\frac{p_{n}[n]_{p_{n},q_{n}}}{[n+1]_{p_{n},q_{n}}}+1 \biggr)+\frac {p_{n}^{n+1}[n]_{p_{n},q_{n}}}{[n+1]_{p_{n},q_{n}}^{2}} \frac{\mu(x)}{1+\mu(x)} \biggr]^{\frac{1}{2}} \biggr\} . \end{aligned}$$

This completes the proof. □

## Pointwise estimation of the operators $L_{n,\mu}^{p,q}$

The aim of this section is to give an estimate concerning the rate of convergence. Here, we take the Lipschitz type maximal function space defined on $F\subset\mathbb{R}_{+}$ (see [30])

$$ \widetilde{E}_{\beta,F}=\biggl\{ \tilde{f}:\sup(1+u)^{\beta}\tilde {f}_{\beta}(u)\leq C\frac{1}{(1+v)^{\beta}}:u\leq0, \mbox{and } v\in F\biggr\} , $$

where *f̃* is a bounded and continuous function on $\mathbb {R}_{+}$, $0<\beta\leq1$ and *C* is a positive constant.

Lenze [31] introduced a Lipschitz type maximal function $f_{\beta}$ as follows:

$$ f_{\beta}(u,v)=\sum_{\substack{ u>0 \\ u\neq v}}\frac{| f(u)-f(v)|}{| u-v|^{\beta}}. $$

### Theorem 4.1

*Let* $L_{n,\mu}^{p,q}$ *be operators defined by* (1.2). *Then*, *for all* $f\in\widetilde{E}_{\beta,F}$, *we have*

$$ \bigl\vert L_{n,\mu}^{p,q}(f;x)-f(x) \bigr\vert \leq C \Bigl( \sqrt{\bigl(\delta_{n}^{\mu}(x) \bigr)^{\beta}}+2 \bigl( \inf\bigl\{ \vert x-y \vert ;y\in F\bigr\} \bigr) ^{\beta } \Bigr) , $$

*where* $\delta_{n}^{\mu}(x)$ *is defined in Theorem* 3.1.

### Proof

Let *F̅* be the closure of *F*. Then there exists $x_{0}\in \overline{F}$ such that $| x-x_{0}|=d(x,F)=\inf\{| x-y| ;y\in F\}$, where $x\in\mathbb{R}_{+}$. Thus we can write

$$ \bigl\vert f-f(x) \bigr\vert \leq \bigl\vert f-f(x_{0}) \bigr\vert + \bigl\vert f(x_{0})-f(x) \bigr\vert . $$

For $f\in\widetilde{E}_{\beta,F}$, we have

$$\begin{aligned}& \bigl\vert L_{n,\mu}^{p,q}(f;x)-f(x) \bigr\vert \\& \quad \leq L_{n,\mu}^{p,q}\bigl( \bigl\vert f-f(x_{0}) \bigr\vert ;x\bigr)+ \bigl\vert f(x_{0})-f(x) \bigr\vert L_{n,\mu}^{p,q}(1;x) \\& \quad \leq C \biggl( L_{n,\mu}^{p,q} \biggl( \biggl\vert \frac{\mu(t)}{1+\mu (t)}-\frac{\mu(x_{0})}{1+\mu(x_{0})} \biggr\vert ^{\beta};x \biggr) + \frac{ \vert \mu (x)-\mu(x_{0}) \vert ^{\beta}}{(1+\mu(x))^{\beta}(1+\mu (x_{0}))^{\beta}}L_{n,\mu}^{p,q}(1;x) \biggr) . \end{aligned}$$

Using the inequality $(u+v)^{\beta}\leq u^{\beta}+v^{\beta}$, we obtain

$$\begin{aligned}& L_{n,\mu}^{p,q} \biggl( \biggl\vert \frac{\mu(t)}{1+\mu(t)}- \frac{\mu (x_{0})}{1+\mu(x_{0})} \biggr\vert ^{\beta};x \biggr) \\& \quad \leq L_{n,\mu}^{p,q} \biggl( \biggl\vert \frac{\mu(t)}{1+\mu(t)}-\frac {\mu(x)}{1+\mu(x)} \biggr\vert ^{\beta};x \biggr) +L_{n,\mu}^{p,q} \biggl( \biggl\vert \frac{\mu(x)}{1+\mu(x)}- \frac{\mu(x_{0})}{1+\mu(x_{0})} \biggr\vert ^{\beta };x \biggr) \\& \quad \leq L_{n,\mu}^{p,q} \biggl( \biggl\vert \frac{\mu(t)}{1+\mu(t)}-\frac {\mu(x)}{1+\mu(x)} \biggr\vert ^{\beta};x \biggr) + \frac{ \vert \mu(x)-\mu (x_{0}) \vert ^{\beta}}{(1+\mu(x))^{\beta}(1+\mu(x_{0}))^{\beta}}L_{n,\mu }^{p,q}(1;x). \end{aligned}$$

Applying Hölder’s inequality, we have

$$\begin{aligned}& L_{n,\mu}^{p,q} \biggl( \biggl\vert \frac{\mu(t)}{1+\mu(t)}- \frac{\mu (x_{0})}{1+\mu(x_{0})} \biggr\vert ^{\beta};x \biggr) \\& \quad \leq L_{n,\mu}^{p,q} \biggl( \biggl( \frac{\mu(t)}{1+\mu(t)}- \frac {\mu(x)}{1+\mu(x)} \biggr) ^{2};x \biggr) ^{\frac{\beta}{2}} \bigl(L_{n,\mu }^{p,q}(1;x)\bigr)^{\frac{2-\beta}{2}} \\& \qquad {}+\frac{ \vert \mu(x)-\mu(x_{0}) \vert ^{\beta}}{(1+\mu(x))^{\beta }(1+\mu (x_{0}))^{\beta}}L_{n,\mu}^{p,q}(1;x) \\& \quad \leq\sqrt{\bigl(\delta_{n}^{\mu} \bigr)^{\beta}}+\frac{ \vert \mu(x)-\mu (x_{0}) \vert ^{\beta}}{(1+\mu(x))^{\beta}(1+\mu(x_{0}))^{\beta}}. \end{aligned}$$

This completes the proof. □

### Corollary 4.2

*For* $F=\mathbb{R}_{+}$, *we have*

$$ \bigl\vert L_{n,\mu}^{p,q}(f;x)-f(x) \bigr\vert \leq C\sqrt {\bigl(\delta_{n}^{\mu}(x)\bigr)^{\beta}}, $$

*where* $\delta_{n}^{\mu}$ *is defined in Theorem* 3.1.

## Other results

### Theorem 5.1

*If* $x\in(0,\infty)\setminus \{ p^{n-i+1}\frac{[i]_{p,q}}{[n-i+1]_{p,q}q^{i}}|i=0,1,2,\ldots,n \} $, *then*

$$\begin{aligned}& L_{n,\mu}^{p,q}(f;x)-f \biggl(\frac{px}{q} \biggr) \\& \quad =- \frac{\mu(x)^{n+1}}{\ell_{n,\mu}^{p,q}(x)} pq^{\frac{n(n-1)}{2}-1} \biggl[\frac{p\mu (x)}{q};\frac{p[n]_{p,q}}{q^{n}}; \bigl(f\circ\mu^{-1}\bigr) \biggr] \\& \qquad {}+\frac{\mu(x)}{\ell_{n,\mu}^{p,q}(x)}\sum_{i=0}^{n-1} \biggl[\frac{p\mu (x)}{q};p^{n-i+1}\frac{[i]_{p,q}}{[n-i+1]_{p,q}q^{i} };\bigl( f \circ\mu^{-1}\bigr) \biggr] \\& \qquad {}\times\frac{1}{[n-i]_{p,q}} p^{\frac{(n-i)(n-i+1)}{2}+1}q^{\frac{i(i-3)}{2}-2} \left [ \textstyle\begin{array}{@{}c@{}} n \\ i\end{array}\displaystyle \right ] _{p,q} \mu(x)^{i}. \end{aligned}$$

### Proof

We have

$$\begin{aligned}& L_{n,\mu}^{p,q}(f;x)-f \biggl(\frac{px}{q} \biggr) \\& \quad =\frac{1}{\ell_{n,\mu}^{p,q}(x)}\sum_{i=0}^{n} \biggl[\bigl( f\circ\mu^{-1}\bigr) \biggl( \frac{ p^{n-i+1}[i]_{p,q}}{[n-i+1]_{p,q}q^{i} } \biggr) -f \biggl(\frac {px}{q} \biggr) \biggr] p^{\frac{(n-i)(n-i-1)}{2}}q^{\frac{i(i-1)}{2}} \left [ \textstyle\begin{array}{@{}c@{}} n \\ i\end{array}\displaystyle \right ] _{p,q} \mu(x)^{i} \\& \quad = -\frac{1}{\ell_{n,\mu}^{p,q}(x)}\sum_{i=0}^{n} \biggl(\frac{p\mu(x)}{q}- \frac{ p^{n-i+1}[i]_{p,q}}{[n-i+1]_{p,q}q^{i} } \biggr) \biggl[\frac{p\mu (x)}{q}; \frac{p^{n-i+1}[i]_{p,q}}{[n-i+1]_{p,q}q^{i} };\bigl( f\circ\mu^{-1}\bigr) \biggr] \\& \qquad {}\times p^{\frac{(n-i)(n-i-1)}{2}}q^{\frac{i(i-1)}{2}} \left [ \textstyle\begin{array}{@{}c@{}} n \\ i\end{array}\displaystyle \right ] _{p,q} \mu(x)^{i}. \end{aligned}$$

Using $\frac{[i]_{p,q}}{[n-i+1]_{p,q}}\bigl [ {\scriptsize\begin{matrix}{} n \cr i\end{matrix}} \bigr ] _{p,q}=\bigl [ {\scriptsize\begin{matrix}{} n \cr i-1\end{matrix}} \bigr ] _{p,q}$, we have

$$\begin{aligned}& L_{n,\mu}^{p,q}(f;x)-f \biggl(\frac{px}{q} \biggr) \\& \quad =-\frac{\mu(x)}{\ell_{n,\mu}^{p,q}(x)}\sum_{i=0}^{n} \biggl[\frac{p\mu (x)}{q};\frac{ p^{n-i+1}[i]_{p,q}}{[n-i+1]_{p,q}q^{i} };\bigl( f\circ \mu^{-1}\bigr) \biggr] \\& \qquad {}\times p^{\frac{(n-i)(n-i-1)}{2}+1}q^{\frac{i(i-1)}{2}-1} \left [ \textstyle\begin{array}{@{}c@{}} n \\ i\end{array}\displaystyle \right ] _{p,q} \mu(x)^{i} \\& \qquad {}+\frac{1}{\ell_{n,\mu}^{p,q}(x)}\sum_{i=1}^{n} \biggl[\frac{p\mu (x)}{q};\frac{ p^{n-i+1} [i]_{p,q}}{[n-i+1]_{p,q}q^{i} };\bigl( f\circ\mu^{-1}\bigr) \biggr] \\& \qquad {}\times p^{\frac{(n-i)(n-i-1)}{2}-(i-n-1)}q^{\frac{i(i-1)}{2}-i} \left [ \textstyle\begin{array}{@{}c@{}} n \\ i-1\end{array}\displaystyle \right ] _{p,q} \mu(x)^{i} \\& \quad =-\frac{\mu(x)}{\ell_{n,\mu}^{p,q}(x)}\sum_{i=0}^{n} \biggl[\frac{p\mu (x)}{q};\frac{ p^{n-i+1} [i]_{p,q}}{[n-i+1]_{p,q}q^{i} };\bigl( f\circ \mu^{-1}\bigr) \biggr] \\& \qquad {}\times p^{\frac{(n-i)(n-i-1)}{2}+1}q^{\frac{i(i-1)}{2}-1} \left [ \textstyle\begin{array}{@{}c@{}} n \\ i\end{array}\displaystyle \right ] _{p,q} \mu(x)^{i} \\& \qquad {}+\frac{\mu(x)}{\ell_{n,\mu}^{p,q}(x)}\sum_{i=0}^{n-1} \biggl[\frac{p\mu (x)}{q};\frac{ p^{n-i}[i+1]_{p,q}}{[n-i]_{p,q}q^{i+1} };\bigl( f\circ\mu^{-1}\bigr) \biggr] \\& \qquad {}\times p^{\frac{(n-i-1)(n-i-2)}{2}-(i-n)}q^{\frac{i(i+1)}{2}-(i+1)} \left [ \textstyle\begin{array}{@{}c@{}} n \\ i\end{array}\displaystyle \right ] _{p,q} \mu(x)^{i} \\& \quad =-\frac{\mu(x)^{n+1}}{\ell_{n,\mu}^{p,q}(x)} \biggl[\frac{p\mu (x)}{q};\frac{p[n]_{p,q}}{q^{n}};\bigl( f \circ\mu^{-1}\bigr) \biggr] pq^{\frac{n(n-1)}{2}-1} \\& \qquad {}+\frac{\mu (x)}{\ell_{n,\mu}^{p,q}(x)}\sum _{i=0}^{n-1} \biggl\{ \biggl[ \frac{p\mu (x)}{q};\frac{ p^{n-i}[i+1]_{p,q}}{[n-i]_{p,q}q^{i+1} };\bigl( f\circ\mu^{-1} \bigr) \biggr] \\& \qquad {}- \biggl[\frac{p\mu(x)}{q};\frac{ p^{n-i+1} [i]_{p,q}}{[n-i+1]_{p,q}q^{i} };\bigl( f\circ \mu^{-1}\bigr) \biggr] \biggr\} p^{\frac{(n-i)(n-i-1)}{2}+1}q^{\frac {i(i-1)}{2}-1} \left [ \textstyle\begin{array}{@{}c@{}} n \\ i\end{array}\displaystyle \right ] _{p,q} \mu(x)^{i}. \end{aligned}$$

By a simple calculation, we have

$$\begin{aligned}& \biggl[\frac{p\mu(x)}{q};\frac{ p^{n-i}[i+1]_{p,q}}{[n-i]_{p,q}q^{i+1} } ;\bigl(f\circ\mu^{-1} \bigr) \biggr] - \biggl[\frac{p\mu(x)}{q};\frac{ p^{n-i+1} [i]_{p,q}}{[n-i+1]_{p,q}q^{i} };\bigl(f\circ \mu^{-1}\bigr) \biggr] \\& \quad = \biggl( \frac{ p^{n-i}[i+1]_{p,q}}{[n-i]_{p,q}q^{i+1} }-\frac{ p^{n-i+1} [i]_{p,q}}{[n-i+1]_{p,q}q^{i} } \biggr) \bigl( f\circ \mu^{-1}\bigr) \\& \qquad {}\times\biggl[ \frac{p\mu (x)}{q};\frac{ p^{n-i+1} [i]_{p,q}}{[n-i+1]_{p,q}q^{i} }; \frac{ p^{n-i} [i+1]_{p,q}}{[n-i]_{p,q}q^{i+1} };\bigl( f\circ \mu^{-1}\bigr) \biggr] \end{aligned}$$

and

$$ \frac{ p^{n-i} [i+1]_{p,q}}{[n-i]_{p,q}q^{i+1} }-\frac{ p^{n-i+1} [i]_{p,q}}{[n-i+1]_{p,q}q^{i} }=[n+1]_{p,q}, $$

we have

$$\begin{aligned} L_{n,\mu}^{p,q}(f;x)-f \biggl(\frac{px}{q} \biggr) =&- \frac{\mu(x)^{n+1}}{\ell_{n,\mu}^{p,q}(x)} \biggl[\frac{p\mu(x)}{q};\frac{p[n]_{p,q}}{q^{n}};\bigl( f\circ \mu^{-1}\bigr) \biggr] pq^{\frac{n(n-1)}{2}-1} \\ &{}+\frac{\mu(x)}{\ell_{n,\mu}^{p,q}(x)}\sum_{i=0}^{n-1} \biggl\{ \biggl[ \frac{p\mu(x)}{q};\frac{ p^{n-i+1} [i]_{p,q}}{[n-i+1]_{p,q}q^{i} };\bigl( f\circ \mu^{-1}\bigr) \biggr] \\ &{}\times \frac{ p^{n-i}[n+1]_{p,q}}{[n-i]_{p,q}[n-i+1]_{p,q}q^{i+1} } \biggr\} p^{\frac{(n-i)(n-i-1)}{2}+1}q^{\frac{i(i-1)}{2}-1} \left [ \textstyle\begin{array}{@{}c@{}} n \\ i\end{array}\displaystyle \right ] _{p,q} \mu(x)^{i}. \end{aligned}$$

This completes the proof. □

## Shape preserving properties

### Theorem 6.1

*Let* $f\in\widetilde{E}_{\beta,\mathbb{R}_{+}}$, *which is a* *μ*-*convex function non*-*increasing on* $\mathbb{R}_{+}$. *Then we have*

$$ L_{n,\mu}^{p,q}(f;x)\geq L_{n+1,\mu}^{p,q}(f;x), \quad n\in\mathbb{N}. $$

### Proof

We have

$$\begin{aligned}& L_{n,\mu}^{p,q}(f;x)-L_{n+1,\mu}^{p,q}(f;x) \\& \quad =\frac{1}{\ell_{n+1,\mu}^{p,q}(x)}\sum_{i=0}^{n} \bigl(f\circ \mu^{-1}\bigr) \biggl( \frac{p^{n-i+1}[i]_{p,q}}{[n-i+1]_{p,q}q^{i}}; \biggr) p^{\frac {(n-i)(n-i-1)}{2}}q^{\frac{i(i-1)}{2}}\left [ \textstyle\begin{array}{@{}c@{}} n \\ i\end{array}\displaystyle \right ] _{p,q} \\& \qquad {}\times\mu(x)^{i}\bigl(p^{n}+q^{n} \mu(x)\bigr) \\& \qquad {}+\frac{1}{\ell_{n+1,\mu}^{p,q}(x)}\sum_{i=0}^{n+1} \bigl(f\circ \mu^{-1}\bigr) \biggl( \frac{p^{n-i+2}[i]_{p,q}}{[n-i+2]_{p,q}q^{i}}; \biggr) p^{\frac {(n-i+1)(n-i+2)}{2}}q^{\frac{i(i-1)}{2}}\left [ \textstyle\begin{array}{@{}c@{}} n+1 \\ i\end{array}\displaystyle \right ] _{p,q}\mu(x)^{i} \\& \quad =\frac{1}{\ell_{n+1,\mu}^{p,q}(x)}\sum_{i=0}^{n} \bigl(f\circ \mu^{-1}\bigr) \biggl( \frac{p^{n-i+1}[i]_{p,q}}{[n-i+1]_{p,q}q^{i}}; \biggr) p^{\frac {(n-i)(n-i-1)}{2}+n}q^{\frac{i(i-1)}{2}}\left [ \textstyle\begin{array}{@{}c@{}} n \\ i\end{array}\displaystyle \right ] _{p,q}\mu(x)^{i} \\& \qquad {}+\frac{1}{\ell_{n+1,\mu}^{p,q}(x)}\sum_{i=0}^{n} \bigl(f\circ \mu^{-1}\bigr) \biggl( \frac{p^{n-i+1}[i]_{p,q}}{[n-i+1]_{p,q}q^{i}}; \biggr) p^{\frac {(n-i)(n-i-1)}{2}}q^{\frac{i(i-1)}{2}+n}\left [ \textstyle\begin{array}{@{}c@{}} n \\ i\end{array}\displaystyle \right ] _{p,q}\mu(x)^{i+1} \\& \qquad {}-\frac{1}{\ell_{n+1,\mu}^{p,q}(x)}\sum_{i=0}^{n+1} \bigl(f\circ \mu^{-1}\bigr) \biggl( \frac{p^{n-i+2}[i]_{p,q}}{[n-i+2]_{p,q}q^{i}}; \biggr) p^{\frac {(n-i+1)(n-i+2)}{2}}q^{\frac{i(i-1)}{2}}\left [ \textstyle\begin{array}{@{}c@{}} n+1 \\ i\end{array}\displaystyle \right ] _{p,q}\mu(x)^{i} \\& \quad =\frac{\mu(x)^{n+1}}{\ell_{n+1,\mu}^{p,q}(x)}q^{\frac {n(n+1)}{2}} \biggl[ \bigl(f\circ \mu^{-1}\bigr) \biggl( \frac{p[n]_{p,q}}{q^{n}} \biggr) -\bigl(f\circ \mu^{-1}\bigr) \biggl( \frac{p[n+1]_{p,q}}{q^{n+1}} \biggr) \biggr] \\& \qquad {}+\frac{1}{\ell_{n+1,\mu}^{p,q}(x)}\sum_{i=1}^{n} \bigl(f\circ \mu^{-1}\bigr) \biggl( \frac{p^{n-i+1}[i]_{p,q}}{[n-i+1]_{p,q}q^{i}}; \biggr) p^{\frac {(n-i)(n-i-1)}{2}+n}q^{\frac{i(i-1)}{2}}\left [ \textstyle\begin{array}{@{}c@{}} n \\ i\end{array}\displaystyle \right ] _{p,q}\mu(x)^{i} \\& \qquad {}+\frac{1}{\ell_{n+1,\mu}^{p,q}(x)}\sum_{i=0}^{n-1} \bigl(f\circ \mu^{-1}\bigr) \biggl( \frac{p^{n-i+1}[i]_{p,q}}{[n-i+1]_{p,q}q^{i}}; \biggr) p^{\frac {(n-i)(n-i-1)}{2}}q^{\frac{i(i-1)}{2}+n}\left [ \textstyle\begin{array}{@{}c@{}} n \\ i\end{array}\displaystyle \right ] _{p,q}\mu(x)^{i+1} \\& \qquad {}-\frac{1}{\ell_{n+1,\mu}^{p,q}(x)}\sum_{i=1}^{n} \bigl(f\circ \mu^{-1}\bigr) \biggl( \frac{p^{n-i+2}[i]_{p,q}}{[n-i+2]_{p,q}q^{i}}; \biggr) p^{\frac {(n-i+1)(n-i+2)}{2}}q^{\frac{i(i-1)}{2}}\left [ \textstyle\begin{array}{@{}c@{}} n+1 \\ i\end{array}\displaystyle \right ] _{p,q}\mu(x)^{i} \\& \quad =\frac{\mu(x)^{n+1}}{\ell_{n+1,\mu}^{p,q}(x)}q^{\frac {n(n+1)}{2}} \biggl[ \bigl(f\circ \mu^{-1}\bigr) \biggl( \frac{p[n]_{p,q}}{q^{n}} \biggr) -\bigl(f\circ \mu^{-1}\bigr) \biggl( \frac{p[n+1]_{p,q}}{q^{n+1}} \biggr) \biggr] \\& \qquad {}+\frac{1}{\ell_{n+1,\mu}^{p,q}(x)}\sum_{i=0}^{n-1} \bigl(f\circ \mu^{-1}\bigr) \biggl( \frac{p^{n-i}[i+1]_{p,q}}{[n-i]_{p,q}q^{i+1}}; \biggr) p^{\frac {(n-i)(n-i-1)}{2}+n}q^{\frac{i(i+1)}{2}}\left [ \textstyle\begin{array}{@{}c@{}} n \\ i+1\end{array}\displaystyle \right ] _{p,q}\mu(x)^{i+1} \\& \qquad {}+\frac{1}{\ell_{n+1,\mu}^{p,q}(x)}\sum_{i=0}^{n-1} \bigl(f\circ \mu^{-1}\bigr) \biggl( \frac{p^{n-i+1}[i]_{p,q}}{[n-i+1]_{p,q}q^{i}}; \biggr) p^{\frac {(n-i)(n-i-1)}{2}}q^{\frac{i(i-1)}{2}+n}\left [ \textstyle\begin{array}{@{}c@{}} n \\ i\end{array}\displaystyle \right ] _{p,q}\mu(x)^{i+1} \\& \qquad {}-\frac{1}{\ell_{n+1,\mu}^{p,q}(x)}\sum_{i=0}^{n-1} \bigl(f\circ \mu^{-1}\bigr) \biggl( \frac{p^{n-i+1}[i+1]_{p,q}}{[n-i+1]_{p,q}q^{i+1}}; \biggr) p^{\frac{(n-i)(n-i+1)}{2}}q^{\frac{i(i+1)}{2}}\left [ \textstyle\begin{array}{@{}c@{}} n+1 \\ i+1\end{array}\displaystyle \right ] _{p,q}\mu(x)^{i+1}. \end{aligned}$$

By a simple calculation, we have

$$\begin{aligned}& \left [ \textstyle\begin{array}{@{}c@{}} n+1 \\ i+1\end{array}\displaystyle \right ] _{p,q}= \frac{[n]_{p,q}[n+1]_{p,q}}{[n-i]_{p,q}[i+1]_{p,q}}\left [ \textstyle\begin{array}{@{}c@{}} n-1 \\ i\end{array}\displaystyle \right ] _{p,q}, \\& \left [ \textstyle\begin{array}{@{}c@{}} n \\ i\end{array}\displaystyle \right ] _{p,q}= \frac{[n]_{p,q}}{[n-i]_{p,q}}\left [ \textstyle\begin{array}{@{}c@{}} n-1 \\ i\end{array}\displaystyle \right ] _{p,q}, \\& \left [ \textstyle\begin{array}{@{}c@{}} n \\ i+1\end{array}\displaystyle \right ] _{p,q}= \frac{[n]_{p,q}}{[i+1]_{p,q}}\left [ \textstyle\begin{array}{@{}c@{}} n-1 \\ i\end{array}\displaystyle \right ] _{p,q}, \end{aligned}$$

we get

$$\begin{aligned}& L_{n,\mu}^{p,q}(f;x)-L_{n+1,\mu}^{p,q}(f;x) \\& \quad =\frac{\mu(x)^{n+1}}{\ell_{n+1,\mu}^{p,q}(x)} q^{\frac {n(n+1)}{2}} \biggl[\bigl( f\circ \mu^{-1}\bigr) \biggl(\frac{p[n]_{p,q}}{q^{n}} \biggr)-\bigl( f\circ \mu^{-1}\bigr) \biggl(\frac{ p[n+1]_{p,q}}{q^{n+1}} \biggr) \biggr] \\& \qquad {}+\frac{1}{\ell_{n+1,\mu}^{p,q}(x)}\sum_{i=0}^{n-1} p^{\frac {(n-i)(n-i-1)}{2}}q^{\frac{i(i+1)}{2}} \frac {[n]_{p,q}[n+1]_{p,q}}{[n-i]_{p,q}[i+1]_{p,q}}\left [ \textstyle\begin{array}{@{}c@{}} n-1 \\ i\end{array}\displaystyle \right ] _{p,q}\mu(x)^{i+1} \\& \qquad {} \times \biggl\{ \bigl( f\circ \mu^{-1}\bigr) \biggl( \frac{ p^{n-i}[i+1]_{p,q}}{[n-i]_{p,q}q^{i+1} }; \biggr)p^{n} \frac{[n-i]_{p,q}}{[n+1]_{p,q}} \\& \qquad {}+\bigl( f\circ \mu^{-1}\bigr) \biggl(\frac{ p^{n-i+1}[i]_{p,q}}{[n-i+1]_{p,q}q^{i} }; \biggr)q^{n-i} \frac{[i+1]_{p,q}}{[n+1]_{p,q}} \\& \qquad {}-\bigl( f\circ \mu^{-1}\bigr) \biggl(\frac{ p^{n-i+1}[i+1]_{p,q}}{[n-i+1]_{p,q}q^{i+1} }; \biggr)p^{n-i}\biggr\} . \end{aligned}$$

By the calculation, $\frac{p[n+1]_{p,q}}{q^{n+1}}-\frac {p[n]_{p,q}}{q^{n}}= ( \frac{p}{q} ) ^{n+1}$, hence we have

$$ \bigl(f\circ \mu^{-1}\bigr) \biggl( \frac{p[n]_{p,q}}{q^{n}} \biggr) - \bigl(f\circ \mu^{-1}\bigr) \biggl( \frac{p[n+1]_{p,q}}{q^{n+1}} \biggr) >0. $$

Since *f* is *μ*-convex, by using [32], we obtain

$$ L_{n,\mu}^{p,q}(f;x)- L_{n+1,\mu}^{p,q}(f;x)>0, $$

where $x\in{}[0,\infty)$ and $n\in\mathbb{N}$.

This completes the proof. □

## Generalization of $L_{n,\mu}^{p,q}$

In this section, we give a generalization of the operators $L_{n,\mu}^{p,q}$ based on $(p,q)$-integers similar to the work done in [30, 33].

Consider

$$\begin{aligned} L_{n,\mu,\gamma}^{p,q}(f;x) =&\frac{1}{\ell_{n,\mu}^{p,q}(x)}\sum _{i=0}^{n}\bigl(f\circ \mu^{-1}\bigr) \biggl( \frac{p^{n-i+1}[i]_{p,q}+\gamma }{\theta _{n,i}} \biggr) \\ &{}\times p^{\frac{(n-i)(n-i-1)}{2}}q^{\frac{i(i-1)}{2}}\left [ \textstyle\begin{array}{@{}c@{}} n \\ i\end{array}\displaystyle \right ] _{p,q}\mu(x)^{i} \quad (\gamma\in\mathbb{R}), \end{aligned}$$

where $\theta_{n,i}$ satisfies the following conditions:

$$ p^{n-i+1}[i]_{p,q}+\theta_{n,i}=b_{n} \quad \mbox{and} \quad \frac{[n]_{p,q}}{b_{n}}\rightarrow1 \quad \mbox{for } n\rightarrow \infty. $$

Note that for $\mu=t$, these operators reduce to [21]. If we choose $\gamma=0$, $q=1$, $p=1$ and $\mu=t$, then we get the Balázs type generalization of *q*-BBH operators [30] given in [33].

### Theorem 7.1

*Let* $p=p_{n}$, $q=q_{n}$, $0< q_{n}< p_{n}\leq1$ *satisfying* (2.5). *Then*, *for any function* $f\in\widetilde{E}_{\beta,\mathbb{R}_{+}}$, *we have*

$$\begin{aligned} \begin{aligned} \lim_{n} \bigl\Vert L_{n,\mu,\gamma}^{p_{n},q_{n}}(f;x)-f(x) \bigr\Vert _{C_{B}} \leq{}&3C\max\biggl\{ \biggl( \frac {[n]_{p_{n},q_{n}}}{b_{n}+\gamma} \biggr) ^{\beta} \biggl( \frac{\gamma}{[n]_{p_{n},q_{n}}} \biggr) ^{\beta}, \\ & \biggl\vert 1-\frac{[n+1]_{p_{n},q_{n}}}{b_{n}+\gamma} \biggr\vert ^{\beta } \biggl( \frac{p_{n}[n]_{p_{n},q_{n}}}{[n+1]_{p_{n},q_{n}}} \biggr) ^{\beta}, \\ &1-2\frac{p_{n}[n]_{p_{n},q_{n}}}{[n+1]_{p_{n},q_{n}}}+\frac{q_{n}[n]_{p_{n},q_{n}}[n-1]_{p_{n},q_{n}}}{[n+1]_{p_{n},q_{n}}^{2}}\biggr\} . \end{aligned} \end{aligned}$$

### Proof

We have

$$\begin{aligned}& \bigl\vert L_{n,\mu,\gamma}^{p_{n},q_{n}}(f;x)-f(x) \bigr\vert \\& \quad \leq \frac{1}{\ell_{n,\mu}^{p_{n},q_{n}}(x)}\sum_{i=0}^{n} \biggl\vert \bigl(f\circ \mu^{-1}\bigr) \biggl( \frac{p_{n}^{n-i+1}[i]_{p_{n},q_{n}}+\gamma}{\theta _{n,i}} \biggr) -\bigl(f\circ \mu^{-1}\bigr) \biggl( \frac {p_{n}^{n-i+1}[i]_{p_{n},q_{n}}}{\gamma +\theta_{n,i}} \biggr) \biggr\vert \\& \qquad {} \times p_{n}^{\frac{(n-i)(n-i-1)}{2}}q_{n}^{\frac{i(i-1)}{2}} \left [ \textstyle\begin{array}{@{}c@{}} n \\ i\end{array}\displaystyle \right ] _{p_{n},q_{n}} \mu(x)^{i} \\& \qquad {}+ \frac{1}{\ell_{n,\mu}^{p_{n},q_{n}}(x)}\sum_{i=0}^{n} \biggl\vert \bigl(f\circ \mu^{-1}\bigr) \biggl(\frac{p_{n}^{n-i+1}[i]_{p_{n},q_{n}}}{\gamma+ \theta_{n,i}} \biggr) -\bigl(f\circ \mu^{-1}\bigr) \biggl(\frac{p_{n}^{n-i+1}[i]_{p_{n},q_{n}}}{[n-i+1]_{p_{n},q_{n}}q_{n}^{i}} \biggr) \biggr\vert \\& \qquad {}\times p_{n}^{\frac{(n-i)(n-i-1)}{2}}q_{n}^{\frac{i(i-1)}{2}} \left [ \textstyle\begin{array}{@{}c@{}} n \\ i\end{array}\displaystyle \right ] _{p_{n},q_{n}} \mu(x)^{i} \\& \qquad {}+ \bigl\vert L_{n,\mu,\gamma}^{p_{n},q_{n}}(f;x) -f(x) \bigr\vert . \end{aligned}$$

Since $f\in\widetilde{E}_{\beta,\mathbb{R}_{+}}$ and by using Corollary 4.2, we can write

$$\begin{aligned}& \bigl\vert L_{n,\mu,\gamma}^{p_{n},q_{n}}(f;x)-f(x) \bigr\vert \\& \quad \leq \frac{C}{\ell_{n,\mu}^{p_{n},q_{n}}(x)}\sum_{i=0}^{n} \biggl\vert \frac{p_{n}^{n-i+1}[i]_{p_{n},q_{n}}+\gamma}{p_{n}^{n-i+1}[i]_{p_{n},q_{n}}+\gamma+\theta_{n,i}}-\frac{p_{n}^{n-i+1}[i]_{p_{n},q_{n}}}{\gamma +p_{n}^{n-i+1}[i]_{p_{n},q_{n}}+\theta_{n,i}} \biggr\vert ^{\beta} \\& \qquad {}\times p_{n}^{\frac{(n-i)(n-i-1)}{2}}q_{n}^{\frac{i(i-1)}{2}} \left [ \textstyle\begin{array}{@{}c@{}} n \\ i\end{array}\displaystyle \right ] _{p_{n},q_{n}} \mu(x)^{i} \\& \qquad {}+ \frac{C}{\ell_{n,\mu}^{p_{n},q_{n}}(x)}\sum_{i=0}^{n} \biggl\vert \frac{ p_{n}^{n-i+1}[i]_{p_{n},q_{n}}}{p_{n}^{n-i+1}[i]_{p_{n},q_{n}}+\gamma +\theta_{n,i}}-\frac{p_{n}^{n-i+1}[i]_{p_{n},q_{n}}}{p_{n}^{n-i+1}[i]_{p_{n},q_{n}}+[n-i+1]_{p_{n},q_{n}}q_{n}^{i}} \biggr\vert \\& \qquad {} \times p_{n}^{\frac{(n-i)(n-i-1)}{2}}q_{n}^{\frac{i(i-1)}{2}} \left [ \textstyle\begin{array}{@{}c@{}} n \\ i\end{array}\displaystyle \right ] _{p_{n},q_{n}} \mu(x)^{i} +C\bigl(\delta_{n}^{\mu} \bigr)^{\frac{\beta}{2}}. \end{aligned}$$

This implies that

$$\begin{aligned}& \bigl\vert L_{n,\mu,\gamma}^{p_{n},q_{n}}(f;x)-f(x) \bigr\vert \\& \quad \leq C \biggl( \frac{[n]_{p_{n},q_{n}}}{b_{n}+\gamma} \biggr) ^{\beta } \biggl( \frac{\gamma}{[n]_{p_{n},q_{n}}} \biggr) ^{\beta} \\& \qquad {}+\frac{C}{\ell_{n,\mu}^{p_{n},q_{n}}(x)} \biggl\vert 1-\frac{[n+1]_{p_{n},q_{n}}}{b_{n}+\gamma} \biggr\vert ^{\beta}\sum_{i=0}^{n} \biggl( \frac{p_{n}^{n-i+1}[i]_{p_{n},q_{n}}}{[n+1]_{p_{n},q_{n}}} \biggr) ^{\beta }p_{n}^{\frac{(n-i)(n-i-1)}{2}}q_{n}^{\frac{i(i-1)}{2}} \left [ \textstyle\begin{array}{@{}c@{}} n \\ i\end{array}\displaystyle \right ] _{p_{n},q_{n}} \mu(x)^{i} \\& \qquad {}+C\bigl(\delta_{n}^{\mu}\bigr)^{\frac{\beta}{2}} \\& \quad =C \biggl( \frac{[n]_{p_{n},q_{n}}}{b_{n}+\gamma} \biggr) ^{\beta} \biggl( \frac{\gamma}{[n]_{p_{n},q_{n}}} \biggr) ^{\beta}+C \biggl\vert 1-\frac{[n+1]_{p_{n},q_{n}}}{b_{n}+\gamma} \biggr\vert ^{\beta}L_{n,\mu,\gamma }^{p_{n},q_{n}} \biggl( \biggl( \frac{\mu(t)}{1+\mu(t)} \biggr) ^{\beta };x \biggr) \\& \qquad {}+C\bigl(\delta_{n}^{\mu}\bigr)^{\frac{\beta}{2}}. \end{aligned}$$

Applying Hölder’s inequality, we get

$$\begin{aligned}& \bigl\vert L_{n,\mu,\gamma}^{p_{n},q_{n}}(f;x)-f(x) \bigr\vert \\& \quad \leq C \biggl( \frac{[n]_{p_{n},q_{n}}}{b_{n}+\gamma} \biggr) ^{\beta } \biggl( \frac{\gamma}{[n]_{p_{n},q_{n}}} \biggr) ^{\beta} \\& \qquad {}+C \biggl\vert 1-\frac{[n+1]_{p_{n},q_{n}}}{b_{n}+\gamma} \biggr\vert ^{\beta }L_{n,\mu,\gamma}^{p_{n},q_{n}} \biggl( \frac{\mu(t)}{1+\mu (t)};x \biggr) ^{\beta} \bigl( L_{n,\mu,\gamma}^{p_{n},q_{n}}(1;x) \bigr) ^{1-\beta }+C\bigl(\delta_{n}^{\mu}\bigr)^{\frac{\beta}{2}} \\& \quad \leq C \biggl( \frac{[n]_{p_{n},q_{n}}}{b_{n}+\gamma} \biggr) ^{\beta } \biggl( \frac{\gamma}{[n]_{p_{n},q_{n}}} \biggr) ^{\beta}+C \biggl\vert 1-\frac{[n+1]_{p_{n},q_{n}}}{b_{n}+\gamma} \biggr\vert ^{\beta} \biggl( \frac{p_{n}[n]_{p_{n},q_{n}}}{[n+1]_{p_{n},q_{n}}}\frac{\mu(x)}{1+\mu (x)} \biggr) ^{\beta} \\& \qquad {}+C\bigl(\delta_{n}^{\mu}\bigr)^{\frac{\beta}{2}}. \end{aligned}$$

This completes the proof. □

## Conclusion

In this paper we have used the $(p,q)$-integers to the Bleimann-Butzer-Hahn operators based on a continuously differentiable function *μ* on $\mathbb{R}_{+}=[0,\infty)$. We have obtained some approximation results on the Korovkin type theorem and computed the rate of convergence by using the modulus of continuity as well as Lipschitz type maximal functions. Further, we investigated the shape preserving properties of these operators.
